# The ADEM2 project: early pathogenic mechanisms of preschool wheeze and a randomised controlled trial assessing the gain in health and cost-effectiveness by application of the breath test for the diagnosis of asthma in wheezing preschool children

**DOI:** 10.1186/s12889-023-15465-6

**Published:** 2023-04-03

**Authors:** Sophie Kienhorst, Moniek H. D. van Aarle, Quirijn Jöbsis, Michiel A. G. E. Bannier, Elin T. G. Kersten, Jan Damoiseaux, Onno C. P. van Schayck, Peter J. F. M. Merkus, Gerard H. Koppelman, Frederik-Jan van Schooten, ADEM2 Consortium, Linda J T M van der Sande, Marieke van Horck, Agnieszka Smolinska, Edward Dompeling

**Affiliations:** 1 Department of Paediatric Pulmonology, Maastricht University Medical Centre, Maastricht, The Netherlands; 2 Department of Paediatric Pulmonology and Pediatric Allergology, Beatrix Children’s Hospital, and GRIAC Research Institute, University Medical Centre Groningen, University of Groningen, Groningen, The Netherlands; 3 Central Diagnostic Laboratory, Maastricht University Medical Centre, Maastricht, The Netherlands; 4 Department of Family Medicine, Care and Public Health Research Institute (CAPHRI), Maastricht University, Maastricht, The Netherlands; 5 Department of Paediatric Pulmonology, Radboud University Medical Centre, Nijmegen, The Netherlands; 6 Department Pharmacology and Toxicology, Faculty of Health, Medicine and Life Sciences, Maastricht University, Maastricht, The Netherlands; 7 Department of Paediatrics, Catharina Hospital Eindhoven, Eindhoven, The Netherlands; 8 Department of Paediatrics, Zuyderland Medical Centre, Heerlen, The Netherlands

**Keywords:** Asthma, Preschool, Wheeze, Diagnosis, Exhaled VOC, Breath test, Biomarkers, Pathogenesis

## Abstract

**Background:**

The prevalence of asthma-like symptoms in preschool children is high. Despite numerous efforts, there still is no clinically available diagnostic tool to discriminate asthmatic children from children with transient wheeze at preschool age. This leads to potential overtreatment of children outgrowing their symptoms, and to potential undertreatment of children who turn out to have asthma. Our research group developed a breath test (using GC-tof–MS for VOC-analysis in exhaled breath) that is able to predict a diagnosis of asthma at preschool age. The ADEM2 study assesses the improvement in health gain and costs of care with the application of this breath test in wheezing preschool children.

**Methods:**

This study is a combination of a multi-centre, parallel group, two arm, randomised controlled trial and a multi-centre longitudinal observational cohort study. The preschool children randomised into the treatment arm of the RCT receive a probability diagnosis (and corresponding treatment recommendations) of either asthma or transient wheeze based on the exhaled breath test. Children in the usual care arm do not receive a probability diagnosis. Participants are longitudinally followed up until the age of 6 years. The primary outcome is disease control after 1 and 2 years of follow-up. Participants of the RCT, together with a group of healthy preschool children, also contribute to the parallel observational cohort study developed to assess the validity of alternative VOC-sensing techniques and to explore numerous other potential discriminating biological parameters (such as allergic sensitisation, immunological markers, epigenetics, transcriptomics, microbiomics) and the subsequent identification of underlying disease pathways and relation to the discriminative VOCs in exhaled breath.

**Discussion:**

The potential societal and clinical impact of the diagnostic tool for wheezing preschool children is substantial. By means of the breath test, it will become possible to deliver customized and high qualitative care to the large group of vulnerable preschool children with asthma-like symptoms. By applying a multi-omics approach to an extensive set of biological parameters we aim to explore (new) pathogenic mechanisms in the early development of asthma, creating potentially interesting targets for the development of new therapies.

**Trial registration:**

Netherlands Trial Register, NL7336, Date registered 11–10-2018.

## Background

Respiratory symptoms, such as wheeze, breathlessness, chronic cough, and sputum production, are very common in young children. Around 40% of all children under the age of 6 suffer from these asthma-like symptoms [1, 2]. Although asthma is the most prevalent chronic disease in children, only the minority (around 30%) of preschool children with recurrent wheeze have persistent wheeze and asthma in later life [1–3]. The majority of wheezing preschool children has transient, viral infection-induced symptoms without an increased risk for asthma and most of the time no need for asthma medication (so called ‘transient wheeze’ or ‘viral wheeze’). At present, there is no proper clinical instrument available that can discriminate between ‘persistent wheeze’ (children with asthma) and ‘transient wheeze’ (children without asthma) at preschool age.

### Prediction of asthma in the guidelines

The prediction of asthma in preschool children with asthma-like symptoms (such as wheeze) has been an important unresolved topic. A reliable asthma diagnosis in preschool wheeze is not possible, as noted by the various (inter)national asthma guidelines [4–7]. These guidelines state that a probability diagnosis based on symptom patterns combined with a careful clinical assessment of family history and physical findings has low positive predictive value. Thus, appropriate treatment and clinical decision-making is hampered in wheezing preschool children.

### Wheezing phenotypes and clinical predictive indices

Whilst no tests diagnose asthma with certainty, various attempts were done to improve an asthma diagnosis in young children. For example, different phenotypes have been described based on triggers of wheeze obtained by clinical history, including episodic (viral) wheeze and multi-trigger wheeze. This was adopted by the European Respiratory Society (ERS) and American Thoracic Society (ATS) taskforce in 2008 [3]. However, accumulating evidence suggested that splitting preschool recurrent wheezing disorders into wheezing phenotypes is an oversimplification, with considerable overlap in symptoms and response to treatment, and is of limited clinical value [8]. Alternatively, clinical indices were developed to classify and predict development of asthma in young children with respiratory symptoms. Various prediction rules, mainly based on clinical parameters, have been developed. Amongst these prediction rules are the (modified) Asthma Predictive Index (API) [9], the Isle of Wright score [10], and the PIAMA risk score [11]. These indices are based on easily obtainable clinical variables. The (modified) API index is increasingly used and mentioned in various guidelines [4]. With the API, young children with a higher risk to develop asthma can be identified based on the age of onset and frequency of wheezing episodes combined with major criteria (parental history of asthma and eczema) and minor criteria (diagnosed allergic rhinitis, wheezing apart from colds, and eosinophilia). Unfortunately, in general the sensitivity of these predictive indices, is low to modest as is their clinical value [12–14]. Moreover, when external validation was performed, these predictive models had only low to modest predictive ability and generalizability (AUC range: 0.62–0.83) [15].

### Consequences of an absent diagnostic instrument for an early diagnosis in wheezing children

In current clinical practice, the absence of a proper diagnostic test for an early asthma diagnosis in young children leads to at least 2 major health problems: 1) children with asthma are frequently underdiagnosed and undertreated; 2) children with transient wheeze are often overtreated with asthma medication.

Considerable underdiagnosis and undertreatment occurs in young children with asthma [16]. Preschool children with asthma and more severe symptoms (having asthma-like symptoms more than twice per week) benefit from maintenance treatment with inhaled corticosteroids (ICS). This improves symptoms, lung function, quality of life, and reduces exacerbation rate and hospital admissions [1]. As a consequence of undertreatment, more children will suffer from uncontrolled asthma with more exacerbations, emergency care visits, hospital admissions, and in the long run even airway remodelling and permanent loss of lung function [17]. Therefore, this undertreatment may lead to reduced quality of life and increased direct and indirect medical costs.

Overtreatment of children with transient wheeze occurs with an increased risk of side effects of ICS and bronchodilators such as reduced linear growth, tremor, and palpitations [1, 16]. Treatment with bronchodilators or ICS might not be very effective in children with viral wheeze, which leads to preventable costs of care and preventable side-effects [1, 17, 18]. Moreover, because of uncertainty of the parents and the primary care physicians, a lot of children with transient wheeze are referred to secondary or tertiary care centres, with additional examinations such as chest X-rays, allergy tests, and treatment with asthma drugs as a consequence.

Therefore, a predictive instrument for an early asthma diagnosis will be of great importance to improve treatment of and care for the large group of young children with wheezing symptoms, and will probably lead to a substantial reduction in the cost of care.

### Volatile organic compounds

Over the past decades, exhaled breath has evolved as a new bodily matrix that has great potential for diagnostic and monitoring purposes [19, 20]. Various research groups demonstrated that exhaled compounds, such as volatile organic compounds (VOCs), can be the base of biomarkers to improve accurate diagnoses and management decisions in pulmonary diseases such as lung cancer and chronic obstructive pulmonary disease. VOCs are an assorted group of carbon-based chemicals that are volatile at room temperature. VOCs are omnipresent in ambient air and once inhaled they are exhaled again in unchanged or metabolised form. At the same time, certain VOCs are endogenously formed in the body during several (patho) physiological processes, such as inflammation, entering the blood stream and exhaled via the lungs [20]. Diseased organs may have a specific profile of VOCs in exhaled breath that distinguish them from healthy organs [19–21]. Although the field of exhaled breath analysis is rapidly growing, VOCs data in children with pulmonary diseases are still limited.

### A predictive breath test for asthma in preschool children

In 2015 we published the results of a longitudinal study in 202 wheezing children, in which we assessed the potential of exhaled breath biomarkers for a paediatric asthma diagnosis, called the ADEM study (Asthma DEtection and Monitoring study) [22]. In this study, we explored whether clinical parameters, genetic information, inflammatory markers in exhaled breath (such as VOCs), and early lung function measurements assessed at preschool age, could help to predict asthma at age 6 in wheezing preschool children. Our most important finding was that a reliable asthma diagnosis could be established in young children (sensitivity 84%, specificity 82%) by combining clinical information (such as family history of asthma, atopic status and symptoms), and a profile of exhaled VOCs [22]. The addition of 9 most predictive exhaled VOCs to a clinical predictive index, significantly improved an asthma diagnosis (AUC, 89%, an increase of 28% compared to using only the asthma predictive index (API)) which persisted in an external validation set. The chemical identity of these 9 VOCs was determined (see Table 1). This is a promising finding, which demonstrates the proof of principle that exhaled breath biomarkers can be used for an early asthma diagnosis.

**Table 1:** the chemical identity of the 9 most predictive VOCs of the ADEM algorithm. BCa 95%CI: Bias Corrected accelerated 95% Confidence Interval [22]

Predicting VOC	Odds ratio	BCa 95% CI	p-value
Acetone	0.93	0.82 to 0.98	< 0.01
Octane	2.91	1.09 to 13.63	< 0.01
2-methylhexane	3.97	1.22 to 126.22	0.01
2,3,6-trimethyloctane	1.51	0.90 to 6.95	0.09
2,6,10-trimethyldodecane	0.98	0.94 to 0.99	0.01
2,4-dimethylpentane	0.79	0.58 to 0.99	0.03
2,4-dimethylheptane	4.25	2.29 to 42.44	< 0.01
2-undecenal	0.08	< 0.01 to 0.53	0.03
2-methylpentane	0.09	< 0.01 to 0.37	< 0.01

### VOCs-sensing techniques in daily clinical practice

In the ADEM study, we used gas chromatography–*time-of-flight*–mass spectrometry (GC-*tof–*MS) to measure exhaled VOCs [22]. Although GC–MS is the gold standard for the identification of VOCs, it is less suitable for clinical application as it is expensive, time consuming and requires extensive technical assistance. Recently, various promising techniques to measure exhaled VOCs such as sensor-based technologies, and selected ion flow tube mass spectrometry (SIFT-MS) technique became of interest [19, 20]. These techniques are cheaper, easier to handle, and provide fast results in comparison to GC–MS. For the application in clinical practice and the implementation in the health care system, a point-of-care breath test based on these faster and cheaper VOCs sensing techniques would be more appropriate.

### Pathophysiological mechanisms

Although the assessment of VOCs is attractive by the non-invasive nature, there is limited understanding of their origin in asthma. VOCs can be formed during various pathophysiological processes, such as airway inflammation and oxidative stress, induced by host and coexisting micro-organisms. Analysing the underlying mechanisms resulting in the formation of predictive exhaled biomarkers such as VOCs, may give insight in the underlying disease pathways leading to the early development of asthma [19, 20]. Understanding pathophysiological mechanisms is the key to improve early diagnosis, monitoring and treatment and maybe even secondary prevention of asthma development. For example, it was found that a disruption of the composition of gut and lung microbiota may be associated with asthma development and that a changed microbiome could be reflected in specific VOCs patterns in exhaled breath. However, the exact role of microbiota and its mechanisms in asthma development are still largely unknown. Also, in the ADEM study multiple genetic risk variants were found to be associated with the development of asthma at age 6 years. For example, there was an interaction between bacterial colonisation of the upper airways, genetic variants in the *TLR*s and *CD14* genes, and the development of asthma at age 6 years [23]. In the same cohort (and replicated in an independent birth cohort) a negative association of the CG/GG-genotype of rs528557 in the *ADAM33* gene with childhood asthma was found, confirming that genetic variation in the *ADAM33* gene may be implicated in the progression of wheeze into childhood asthma [24]. In an integrative genomic approach, data suggested that *ICAM-1* was likely to be involved in the development of childhood asthma [25]. Although these data provide valuable insight in the development of childhood asthma, the cumulative predictive power of genetic risk variants in polygenic risk scores is limited [26]. Additional layers of ‘omic’ data, such as epigenetics, may be more powerful as predictive biomarkers.[26] Epigenetics refers to DNA changes that regulate gene expression without altering the DNA sequence. A recent study showed the high diagnostic accuracy of nasal DNA methylation to diagnose allergic asthma in school aged children in a cross-sectional design [27]. Moreover, blood DNA-methylation at 14 CpG sites was associated with childhood asthma as early as the age of 4 years. These whole blood signatures were driven by large DNA-methylation differences within sorted eosinophils, which has promising diagnostic potential [28].

### Objectives of the new study: the ADEM2 study

The previous ADEM study generated a lot of insight in the diagnostic potential of VOC patterns in exhaled breath with respect to an early diagnosis of asthma, and in multiple potential underlying pathophysiological mechanisms leading to the development of asthma. However, since then, many new questions arose. For example with regards to the feasibility of bringing the assessment of VOCs in exhaled breath into daily clinical practice, and with respect to the impact an early diagnosis of asthma will have on wheezing preschool children and their parents. Also, recent developments in the evolving field of multi-omics generated new approaches to investigate the early pathogenesis of asthma. Therefore, a new randomised controlled trial and prospective study in wheezing preschool children was designed, the ADEM2 study.

The primary objective of the ADEM2 study is to prove health gain and reduction in costs by application of a point-of-care (POC) breath test for an early asthma diagnosis in preschool children. A multicentre randomised controlled trial (RCT) in 220 wheezing preschool children will be performed. Children will be randomised into an intervention group (*n* = 110) in which the doctors and parents will be informed about the predicted asthma diagnosis provided by the breath test, or into a usual care group (*n* = 110) in which all parties are masked for the predicted diagnosis. The second objective is to further develop and validate a reliable, non-invasive point-of-care breath test. In a prospective observational study we will assess feasibility, accuracy and reproducibility of the innovative VOC techniques (e.g. SIFT-MS and VOC sensors) in comparison with the gold standard breath test (GC-tof–MS), for an early asthma diagnosis in wheezing preschool children. The third and final objective of the ADEM2 project is to combine metabolomic, immunological, (epi)genomic, transcriptomic, and microbiome data to unravel potential important pathways for asthma development. Moreover, the utility of predictive, multi-omic testing of childhood asthma in wheezing pre-school children will be explored. This knowledge can offer clues for novel diagnostic tests and proper or even revised treatment (e.g. microbiota-based therapy) in wheezing children.

### Hypotheses of the ADEM2 study

The hypothesis of the clinical trial of the ADEM2 study is that application of the breath test in preschool children with asthma-like symptoms will result in important health gain and reduction of costs of care. In children with asthma, we expect that an early diagnosis by the breath test will result in more targeted and better treatment. This will facilitate better asthma control, improved lung function, less exacerbations, less hospital admissions, improved quality of life, and probably less airway remodelling. In children with transient wheeze, we hypothesise that an early diagnosis will diminish uncertainty of parents and treating doctors, with less referrals, less additional examinations (X-rays, allergy tests, microbial cultures or viral PCR tests), and less ineffective treatment (antibiotics, corticosteroids, or antihistamines). Parents will be reassured by the early diagnosis of transient wheeze, which will probably increase quality of life and decrease medical consumption. In both cases, cost savings are expected because of a proper diagnosis by the breath test due to significantly reduced hospital based care and medication use in children with transient wheeze, and prevention of undertreatment related sequela in children with asthma.

Our hypothesis of the observational cohort study with respect to the feasibility, reproducibility and accuracy of the alternative VOC-sensing techniques is that innovative techniques such as SIFT-MS are not inferior to the current golden standard GC–MS. We also hypothesise that new (combinations of) biomarkers will be found that improve the diagnostic accuracy to identify the wheezing preschool child developing asthma, such as epigenetic signatures of blood eosinophils, microbiome data of both nasopharyngeal swabs and faecal samples, whole RNA sequencing on blood, gene-expression of relevant asthma genes, and immunological markers.

## Methods

### Study design

The proposed study is a combination of a multi-centre, parallel group, two arm, randomised controlled trial and a multi-centre longitudinal observational cohort study. All children that participate in the RCT will also contribute to the observational cohort study.I

### Study setting

In order to achieve a good mixture of disease variability and severity in the study population (to maximise the external validity of the study results), the trial will be conducted in primary care practices and in the paediatric wards and outpatient departments of several general hospitals and university hospitals in the Netherlands. Preschool children aged 2 or 3 years will be recruited. The participating recruiting primary care practices are located in the southern and northern region of the Netherlands. The participating general hospitals are located throughout the country, as well as the participating university hospitals (Maastricht University Medical Centre, University Medical Centre Groningen, and Radboud University Medical Centre).

The healthy children that contribute to the observational cohort study will be recruited at day-care centres, via centres for youth health care, and by means of primary care practices, and (social) media.

### Eligibility criteria

The proposed study protocol includes both wheezing preschool children and healthy preschool children. In the RCT only wheezing preschool children will participate, whereas in the longitudinal cohort study both wheezing and healthy preschool children participate.

### Inclusion criteria

- Wheezing children: age between 2 and 4 years old and presence of two or more objectified (by a physician or nurse) episodes of wheezing and shortness of breath
- Healthy children: age between 2 and 4 years old

### Exclusion criteria

- Wheezing children: presence of chronic and/or inflammatory disease other than asthma (e.g. inflammatory bowel disease, auto-immune disorders, cardiac disease, congenital lung disease, kidney or liver disease), prematurity < 35 weeks gestational age, postnatal need for oxygen, CPAP, non-invasive or invasive ventilation, or mental disability.
- Healthy children: a history of asthma or wheeze, other chronic and/or inflammatory disease other than asthma (e.g. inflammatory bowel disease, auto-immune disorders, cardiac disease, congenital lung disease, kidney or liver disease), prematurity < 35 weeks gestational age, postnatal need for oxygen, CPAP, non-invasive or invasive ventilation or mental disability.

Children with a recent course of systemic corticosteroids or antibiotics are not excluded from participation, but the baseline visit will be postponed until at least one month after the treatment.

### Intervention

The intervention in the RCT consists of providing a probability diagnosis of either asthma or transient wheeze based on the earlier developed breath test of the ADEM study [22]. Based on this probability diagnosis, disease-specific recommendations on treatment and follow-up will be provided to the parents and treating physicians. At the start of the study, exhaled breath will be collected and sent to the central laboratory in the Maastricht UMC + for analysis on a GC-*tof*–MS. After analysis, the chromatogram is compared with our central database (based on the results of the ADEM study [22]) in order to determine whether the newly taken breath sample fits an asthmatic or a transient wheeze pattern of the selected VOCs. Parents and doctors of children allocated to the intervention group will receive the test result (probability diagnosis “asthma” or “transient wheeze”) within three months after the start of the study. In the children with a test result of “transient wheeze” in the intervention group, a restrictive policy towards use of asthma medication and referral to specialist care will be advised. In children with a test result of “asthma”, treatment with asthma medication according to international guidelines will be advised [29]. Parents and doctors of patients allocated to the usual care group will receive the test result at the end of the study at the age of 6 years. The management of preschool children with recurrent wheezing will be in accordance with national and international guidelines [29–31]. Both in the intervention and control group, treating doctors are at any time free to prescribe asthma medication, antibiotics or other drugs they judge as clinically necessary or meaningful.

### Outcomes

#### Randomised controlled trial

- Primary outcome: difference between the intervention group and the usual care group in the percentage of well controlled asthma-like symptoms after 1- and 2-year follow-up. The percentage well controlled asthma-like symptoms will be based on the validated TRACK questionnaire.
- Secondary outcomes: differences after one year and at the end of the study between the intervention group and usual care group will be assessed with respect to:
  - Number of exacerbations
  - Lung function (spirometry) at 6 years of age
  - Airway resistance with forced oscillation technique
  - Quality of life of children and their parents
  - Pharmacotherapy (frequency and dosage of used medication)
  - Growth velocity over 12, 24 and 36 months (cm/y), change in height SD scores over 12, 24 and 36 months
  - Patient reported side-effects of medication
  - Healthcare resource use and –costs (standard and extra clinical visits, hospital admissions, referrals, laboratory tests, imaging tests)
  - Costs outside healthcare (over-the-counter drugs)
  - Absence of school and work (parents);

### Prospective cohort study

- Secondary outcomes
  - The sensitivity and specificity of new VOC sensing techniques (e.g. SIFT-MS and VOC sensors) for a diagnosis of asthma or transient wheeze in preschool children. Assessment at the start of the study in relation to the current gold standard in breath research (GC–MS) and at the age of 6 years in relation to the final diagnosis.
  - Identification of other potential discriminating biological parameters (such as allergic sensitisation, immunological markers, epigenetics, transcriptomics, microbiome) between asthma, transient wheeze, and healthy controls, and the subsequent identification of underlying disease pathways and relation to the discriminative VOCs in exhaled breath. Assessment of data collected at the start of the study and at the end in relation to the final diagnosis at the age of 6 years.
  - Identification of discriminative exhaled VOCs (based on GC-*tof*–MS analysis) between children with asthma, children with transient wheeze and healthy children. Assessment of data collected at the start of the study and at the end of the study in relation to the final diagnosis at the age of 6 years.

### Participant timeline

**Fig. 1:**
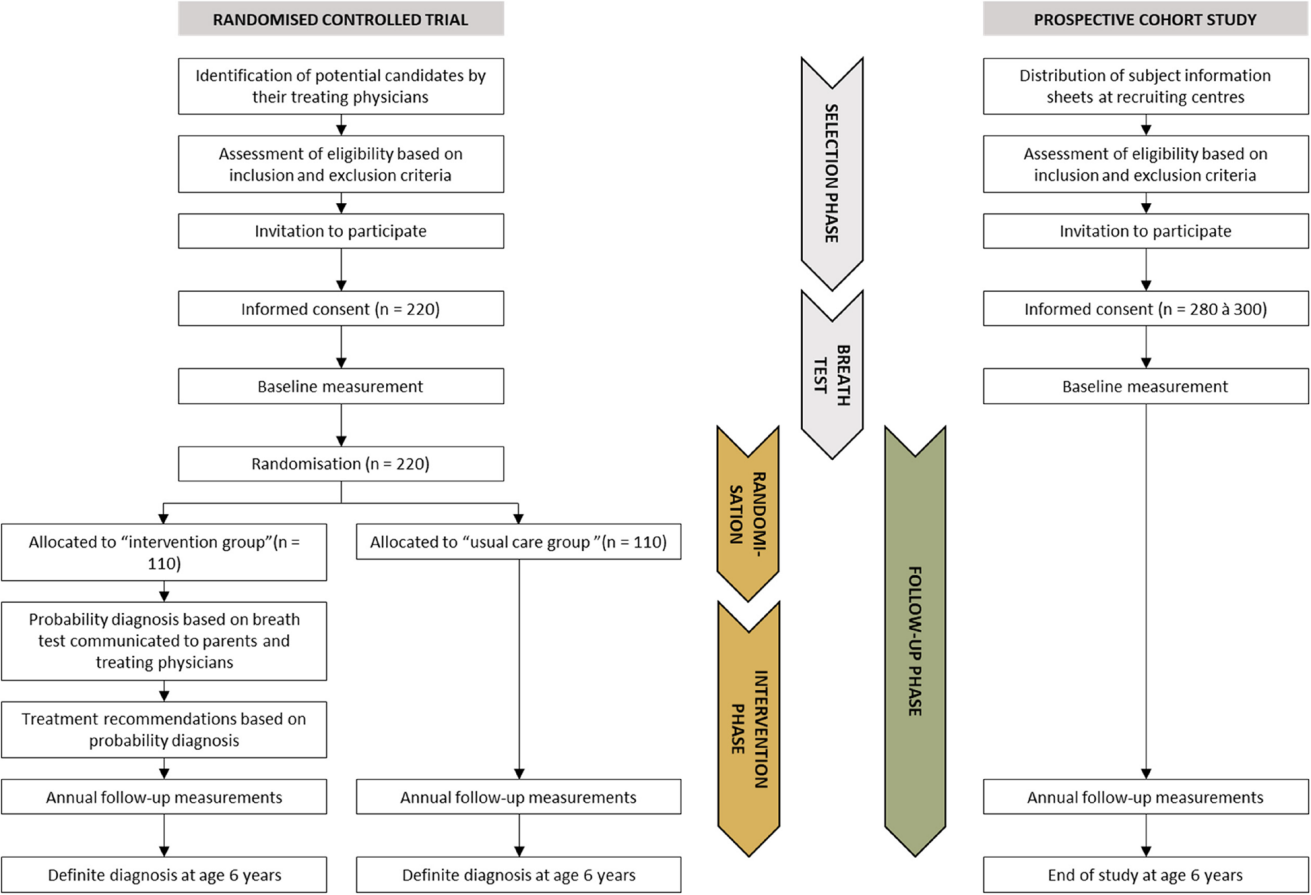
Flow of participants

**Fig. 2:**
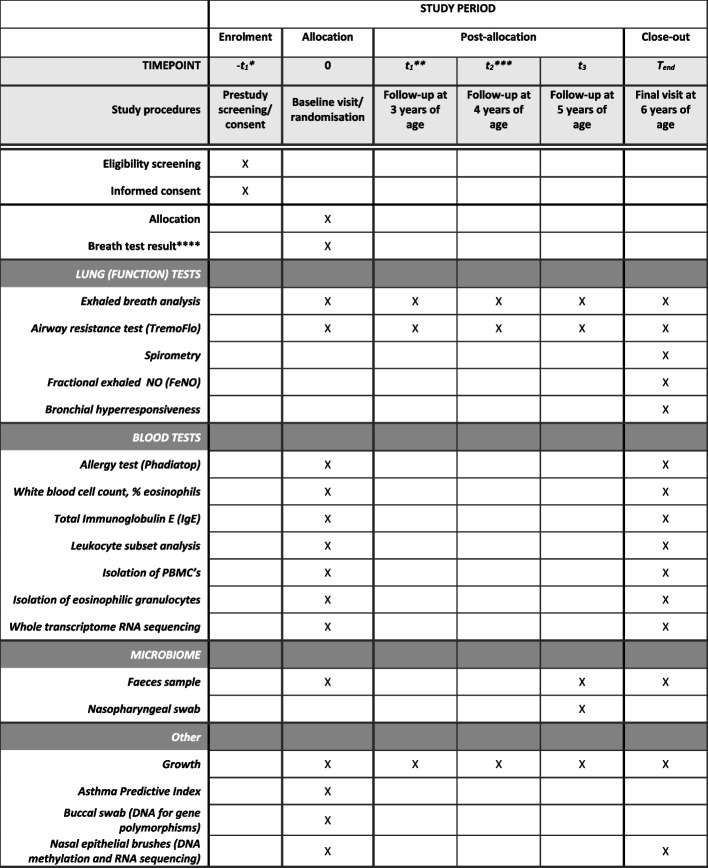
Schedule of enrolment, interventions, and assessments. * Children can enrol the study at 2 or 3 years of age. ** Only applicable for patients enrolling the study at 2 years of age_._ *** Applicable for patients enrolling the study at 2 or 3 years of age. **** Applicable to those patients randomised into the treatment group. PBMC Peripheral blood mononuclear cells

**Fig. 3:**
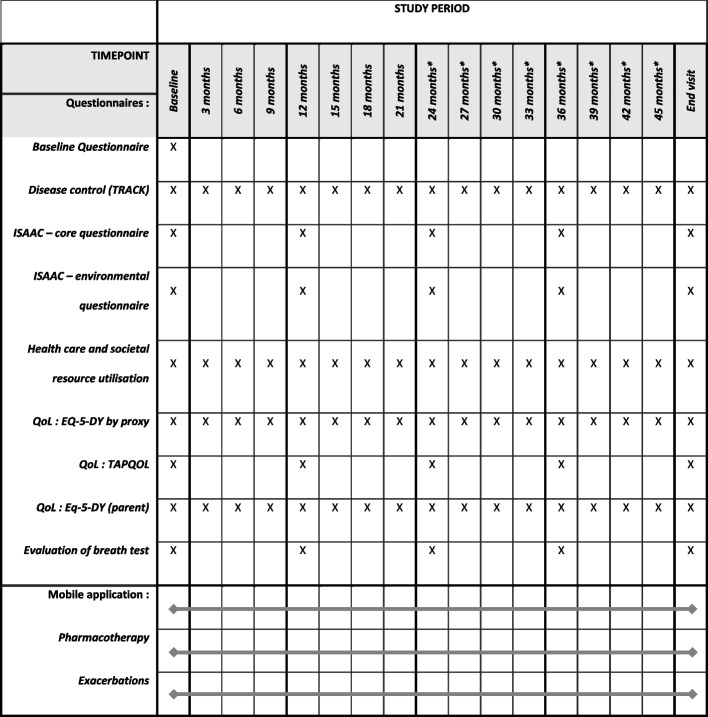
Schedule of questionnaires. *QoL* Quality of life. * Applicable for children enrolling the study at 2 or 3 years of age

Both wheezing participants and healthy participants follow the same timeline (Fig. 1). Participants enrol in the study, after informed consent, at the age of 2 or 3 years old. At the baseline visit, the wheezing children that participate in the RCT will be randomised into either the intervention group or the usual care group. All children included in the study will perform the study-related procedures, including the breath test, at the baseline visit (see section “data collection” and Fig. 2). The children that will be allocated to the intervention arm of the RCT will receive their probability diagnosis and corresponding treatment advice within three months of the baseline visit. All children will be invited for the annual, study-related hospital visits until the age of 6. The performed study procedures at each visit are illustrated in Fig. 2. All parents will receive online questionnaires prior to the annual visit (a list of the questionnaires is provided in Fig. 3). Parents of children that participate in the RCT will also receive questionnaires on disease control and healthcare related expenditures with a 3-month interval. At the age of 6, a final diagnosis will be made based on respiratory symptoms, use of medication, and objective lung function measurements as reported previously [22].

### Sample size

*Randomised controlled trial* With a presumed percentage of well-controlled preschool children with asthma-like symptoms of 20% in the usual care group (based on reference [32, 33]), and of 40% in the intervention group, 91 patients in both groups are needed to detect this difference with an alpha of 0.05 and a power of 80%. Taking a dropout rate of 10% into account, we aim to include a total number of 220 preschool children with asthma-like symptoms.

*Prospective cohort study* For studies assessing the relationship between microbiome, transcriptomics, epigenetics, and atopic outcomes like asthma, a sample size of 70–80 is considered adequate [34, 35].

### Recruitment

Wheezing participants will be recruited at the participating primary care centres and participating hospitals. Potential candidates will be identified by their treating physicians and (specialised) nurses at the primary care practices, outpatient departments, paediatric wards or emergency departments during the recruitment and enrolment phase of the study. Subject information sheets on the study will be handed out to these patients and the parents will be asked to contact the research team for more information about participating in the study.

Healthy participants will be recruited through advertisement and distribution of subject information sheets to all parents of 1- to 4-year old toddlers at day-care centres, via centres for youth health care, via general practitioners practices and during pre-operative screening at the outpatient department of the paediatric anaesthesia department of Maastricht University Medical Centre. Also a variety of (social) media will be used for open advertisement.

All recruiting materials and subject information sheets have prior approval of the institutional Medical Research Ethics Committee (MREC).

### Allocation and blinding

The wheezing preschool children that participate in the RCT will be randomly assigned (1:1), with a secure computer-generated block randomisation procedure (block size of 6), into a usual care group and an intervention group. The randomisation will be organised by the Clinical Trial Centre Maastricht (CTCM) and MEMIC (centre for data and information management at the Faculty of Health, Medicine and Life Sciences of Maastricht University and MUMC +). Randomisation is stratified per measurement centre. The participants, treating physicians and the researchers or research assistants involved in conducting the baseline visit are not blinded to the results of the randomization procedure. The assessment of the primary outcomes and secondary outcomes after one year and at the end of the study will be done by researches who were not involved in the recruitment and randomization procedures. These researchers will be blinded towards the allocation in randomisation group and (final) diagnosis of the participants.

### Data collection

During the annual visits, various study procedures will be conducted. Each centre in which the study visits take place (the “measurement centres”) has a dedicated team consisting of one or two research nurses, and one or two p that execute all study-related procedures. All study personnel will be trained in the study requirements and study procedures. Standard Operating Procedures (SOPs) will be provided to all study sites to enhance data quality and reduce variability in measurements as much as possible.

Parents will be instructed that their children should refrain from eating, brushing teeth, using inhalation medication, and moderate to severe exercise as much as possible within 60 min prior to the visit. Drinking of water is allowed prior to the tests. Prior to the annual visits, electronic questionnaires will be sent to the parents. In the RCT study group, a subset of these questionnaires will also be sent at three monthly intervals. The parameters that are measured are listed in Fig. 2.

A web-based study management system Ldot (https://nl.ldot.nl/) will be used to monitor the study logistics and guard the process of the research project. Ldot enhances participant retention by sending both automatically generated reminders for study personnel to contact patients, and automatically generated reminders to study participants to attend planned visits (both by e-mail and SMS messages). Also, periodic communications via newsletters and a website will be provided to parents, recruiting doctors, and treating doctors of participating patients.

### Volatile Organic Compounds

**Fig. 4:**
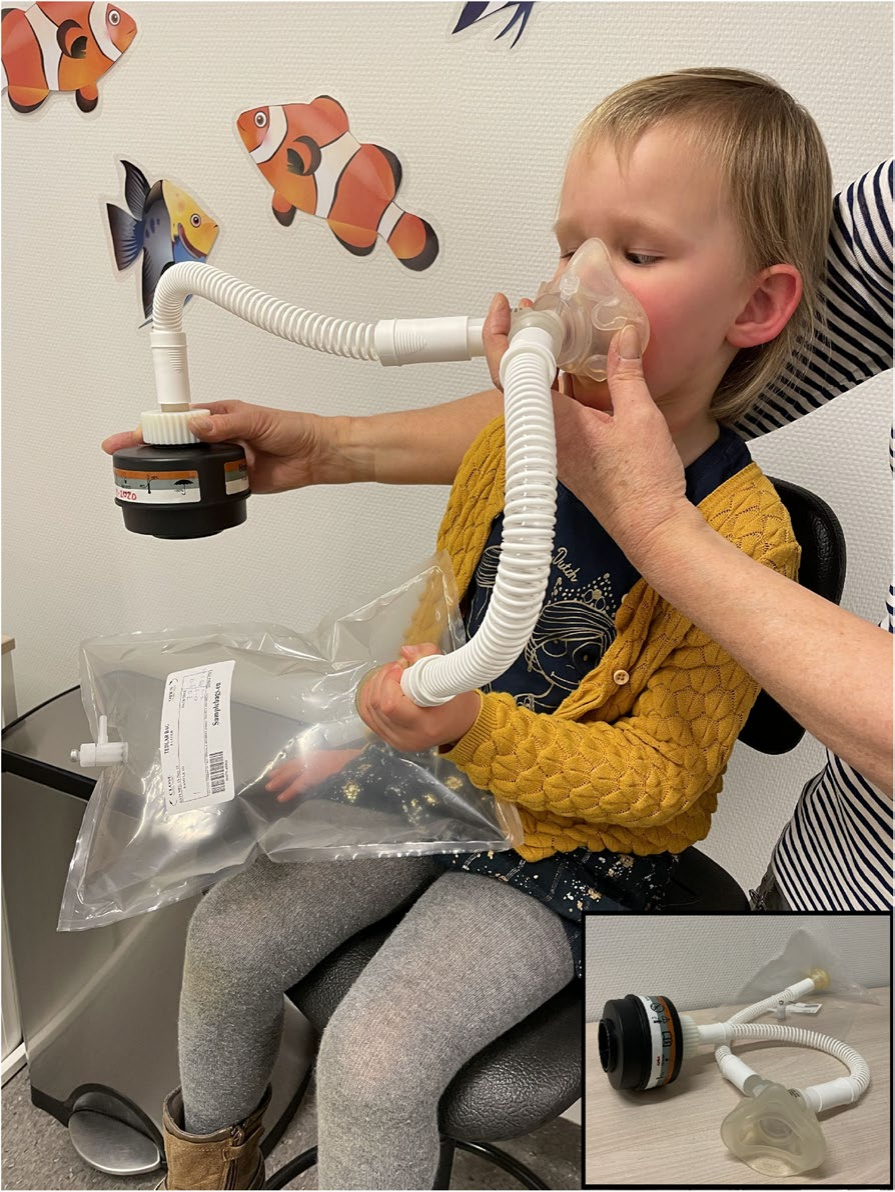
Breath collection in a child using a tailored breath sampling system (see insert)

*Breath collection:* Breath samples will be collected by using a tailored breath sampling system for children developed by our department (Fig. 4). The children breathe tidally and without resistance through a silicone oro-(mouth) mask (Hans Rudolph, Inc., Kansas, USA), separating nasal and bronchial expired air, and connected to a Y-shaped, non-rebreathing two-way valve system (Hans Rudolph, Inc., Kansas, USA) [24]. At the inhalation port of the two-way valve, a VOC-filter (Combined filter A2B2P3, Honeywell, United States) is attached. This ensures inhaling environmental air free from exogenous VOCs. On the expiratory port of the valve a custom made 3-L polycarbonate bag (Tedlar® bag, samplebags.eu, The Netherlands) will be connected to collect the exhaled breath. The child will first be asked to breathe tidally for 3 min to ensure that the entire lung capacity is refreshed with environmental-VOC-free air. After 3 min the sampling bag will be attached to the exhalation port. After the bag is filled up to a maximum of 80%, the bag will be disconnected from the sampling device.

*SIFT-MS analysis:* the sampling bag will be connected to the Syft Voice 200 Ultra (Syft technologies, Christchurch, New Zealand) for SIFT-MS analysis of the breath. The instrument will be applied in full spectral mass scan mode in the mass-to-charge ratio (m/z) range of 15–250 amu for all three precursor ions (H3O^+^, NO^+^, O2^+^). Three acquisition repeats will be performed in a single run and the full scan data (ion counts per second) will be averaged over the three repeats for each m/z value. The SIFT-MS measurement uses approximately 60 ml of exhaled breath.

*GC-tof–MS analysis* the remaining breath in the sampling bag will be emptied across a stainless steel, two-bed sorption tube filled with Carbograph 1 TD/Carbopack™ X (Markes International, Llantrisant, UK) for rapid adsorption and stabilisation of volatile compounds. The tubes are airtight capped and stored at 4 °C until analysis. During the analysis, VOCs are first released from the tube using thermal desorption (Unity desorption unit; Markes International) at 270 degrees Celsius. In the next step, 25% of the mixture of vapour is loaded onto a cold (5 °C) sorption trap, while the remaining 75% of the mixture is recollected into an identical sample tube. The vapour mixture was then reloaded from trap into the gas chromatography time-of-flight mass spectrometry (Tempus Plus; ThermoFischer Scientific) (GC-tof–MS) analysis. The temperature of the GC was programmed as follows: first 40 °C for 5 min then it was increased by 10 °C every minute until 270 °C is reached. This temperature is maintained for 5 min. Electron ionisation at 70 eV was used with 5 Hz scanning rate over a range of m/z 35–350 [36]. The pre-processing of the raw GC-*tof*–MS spectra consists of noise removal, baseline correction, alignment, and peak detection. Thereafter, complementary compounds in different samples are linked, based on similarity of retention times and mass spectra. The area under the peak will be calculated for each compound. To make the spectra comparable, normalisation to the total area will be performed [37].

### Lung function

*Airway resistance test:* the airway resistance will be measured annually by means of the TremoFlo C-100™(Thorasys, Montreal, Canada) using the flow-oscillation technique (http://thorasys.com). The children will sit upright with their head in a neutral position and the researcher standing behind them to manually support the cheeks to minimise the upper airway shunt. The child will be asked to breathe quietly with the mouthpiece into his or her mouth, while making a seal with the lips around the mouthpiece and wearing a nose clip. The measurements will be repeated until at least three, artefact free measurements are completed. Thereafter, 300 µg of salbutamol will be inhaled via the Aerochamber®. After 15 min, the airway resistance measurements are repeated to assess the reversibility to a beta-2 agonist.

*Spirometry, bronchial NO and bronchial hyperresponsiveness:* additional lung function tests are performed during the final visit at age six years in order to make a definite asthma diagnosis in all participating children. These tests are performed and selected according to the ERS clinical practice guidelines for the diagnosis of asthma in children [38]. Spirometry and bronchodilator reversibility (BDR) testing is performed in which the highest forced expiratory volume in one second (FEV1), forced vital capacity (FVC), and maximal expiratory flow at 50% FVC (MEF50) of three technically satisfactory MEFV curves will be used for analysis. Also, in all children the fraction of exhaled nitric oxide (FeNO) will be measured with an online single breath technique with constant expiratory flow (NIOX VERO ®, Circassia AB, Oxford, UK). The Fractional exhaled NO (FeNO) value will be expressed as parts per billion. Only in a subset of patients in which a conclusive asthma diagnosis cannot be made based on spirometry, BDR and FeNO, a direct bronchial challenge test will be performed by administrating aerosols of methacholine.

### Microbiome

*Nasopharyngeal swab:* a nasopharyngeal swab will be taken at the age of 5 years. A sterile nylon flocked swab (FLOQSwabs®, COPAN, CA, USA) will be used. An aliquot of these swabs will be stored in Universal Transport Medium (UTM) at -80°C until used for determination of the microbiota of the lower airways.

*Faecal sample:* faecal samples of the children will be collected at baseline, at the age of 5 years and at the age of 6 years. These samples will be stored at -80°C and eventually used for microbiome analysis.

### Buccal swab

Isohelix Buccal swabs with RapiDri™ pouch will be used to sample buccal cells for isolating DNA at the baseline visit. The extracted DNA is used to study gene polymorphisms in selected candidate genes. The inclusion of genes for SNP analysis is based on the following criteria: association with asthma based on biomedical literature, a functional difference between the variant allele and the wild-type allele, and a minor allele frequency of at least 5% in the (asthmatic) population.

### Nasal epithelial brush

Nasal epithelial cells will be collected at the baseline visit by brushing nylon flocked swabs (FLOQSwabs®, COPAN, CA, USA) against the lateral side of the inferior turbinate of both nostrils. Two swabs will be transferred into sterile National Lab Cryovials, and two swabs will be transferred into sterile National Lab Cryovials filled with RNAlater™ stabilisation solution. All cryovials will be stored at -80°C until DNA and RNA extraction and subsequent determination of DNA methylation and RNA-sequencing.

### Venous blood sample

Six millilitres of venous blood will be sampled at baseline visit and at the end visit. One to two hours prior to the blood puncture, lidocaine 1% gel with 4 × 4 cm plaster will be applied. This blood will be used for.

- allergy testing: total immunoglobulin E (IgE) and determination of specific IgE antibodies to inhalant allergens (ImmunoCAP allergens gx3 (grass pollen), tx9 (tree pollen), wx3 (weed pollen), mx1 (moulds), d1 (house dust mite), e1 (cat dander), e5 (dog dander) (Phadiatop test; Phadia, Uppsala, Sweden)
- white blood cell count and absolute number of eosinophils will be determined
- leukocyte subset analysis by flow cytometry: Extended phenotyping will be performed to evaluate B-cell maturation and differentiation (CD19, CD27, CD38, and IgD), and T-cell maturation and differentiation (CD3, CD4, CD8, CD28, CD45RA and CD127). These panels enable the distinction of pro- and anti-inflammatory lymphocyte subsets, the degree of memory formation as a marker for pathogen exposure, and early senescence. In addition, monocyte subsets (classical and non-classical) and dendritic cell subsets (myeloid and plasmacytoid dendritic cells) will be evaluated (CD11c, CD14, CD16, CD123, HLA-Dr, BDCA-2, and BDCA-3)
- isolation of peripheral blood mononuclear cells (PBMC’s): PBMC’s will be stored in liquid nitrogen for in vitro activation with distinct stimuli followed by analyses of the produced cytokine repertoire.
- isolation of eosinophils: In a subset of children (80 children with wheeze, 40 healthy controls) peripheral blood eosinophils will be isolated by FACS sorting. Blood eosinophils will be isolated from 2 ml of EDTA blood using an adapted FACS sorting strategy based on Mori et al. within 24 h of blood sampling, sorting Siglec8 + and CD193 + cells [39]. From sorted eosinophils, we will isolate DNA with the DNeasy blood and tissue kit (Qiagen, Venlo, the Netherlands) and investigate DNA-methylation using the Infinium Human Methylation EPIC Bead Chip array (Illumina, San Diego, USA).
- whole transcriptome RNA sequencing for gene expression of markers of inflammation and oxidative stress. One millilitre of venous blood will be transferred into sterile National Lab Cryovials. Invitrogen™ RNA*later*™ Stabilization Solution will be added to ensure immediate RNase inactivation and RNA stabilization within cells. All cryovials will be stored at -80°C until RNA extraction and subsequent RNA-sequencing.

### API and modified API (mAPI)

The API and mAPI (based on parental asthma, eczema, allergic rhinitis, wheezing apart from colds, and atopy) will be assessed at baseline [22].

### Questionnaires

Parents will be asked to complete several questionnaires during the study to assess asthma control, quality of life, and utilisation of health care and societal resources [40–42]. The questionnaires will be provided as e-version and will be sent to the parents two weeks before the annual clinical visits. Two questionnaires will be sent at a three-monthly interval: the questionnaires on asthma control (TRACK) and on utilisation of health care and societal resources.

*TRACK* Parents will be asked to complete the TRACK questionnaire at a 3-month interval. The TRACK questionnaire is a validated questionnaire on asthma control specifically developed for use in this age group, independent of the diagnosis. A score of 80 or more is defined as well controlled disease. The TRACK score is sensitive and reliable, and an increase of 10 points was found to be the ‘minimally important difference’ [43].

*ISAAC questionnaire (Core Questionnaire and Environmental Questionnaire)* The Core Questionnaire and the Environmental Questionnaire have been developed by the ISAAC steering Committee [44]. This questionnaire is used in this study to assess asthma, allergic rhinoconjunctivitis and eczema in all participants and to assess a variety of environmental factors.

*EQ-5D-Y (proxy version)* The EQ-5D-Y questionnaire is a validated child-friendly version of the EQ-5D on quality of life that comprises the following five dimensions with three answer levels (“no problems,” “some problems,” and “a lot of problems”): mobility, looking after myself, doing usual activities, having pain or discomfort and feeling worried, sad or unhappy. It also includes a visual analogue scale (VAS), which gives an overall assessment of the child’s health status in a scale from 0 (worst imaginable health state) to 100 (best imaginable health state). In the proxy version, the caregiver is asked to rate the child’s health-related quality of life (HRQoL). The EQ-5D-Y proxy-version has been tested in children as from 4 years of age [45], which revealed that some domains (e.g. self-care) were sometimes perceived by the parents as not suitable to young children. However, no alternative preference-based measure is currently available or has a validated proxy-version for use in preschool children.

*EQ-5D-5L* Quality of life of one of the parents will be assessed annually by the Eq-5D-5L [46]. The EQ-5D-5L comprises the same dimensions as the above mentioned EQ-5D-5L, but each dimensions has 5 levels (no problems, slight problems, moderate problems, severe problems and extreme problems). It also includes a vertical visual analogue scale to record the parents’ self-rated health.

*TAPQOL* The TAPQOL measures parent’s perceptions of HRQoL in preschool children. The questionnaire was developed for children between 9 months and 6 years old. The TAPQOL will be completed by one of the parents at baseline, and at 1 and 2 years of follow-up [42].

*Utilisation of health care and societal resources* a self-composed questionnaire to assess the number of days the child is not able to go to school/day-care, the number of days the parents are not able to work, the resource use within health care (control visits, emergency visits, hospital admission, visits to the doctor, lung function tests and other diagnostic procedures, medication) and ‘outside health care’ (over the-counter medication). This questionnaire will be completed at 3 months intervals by parents.

### Cost-effectiveness

Cost-effectiveness will be calculated as the incremental costs per child with well-controlled disease (based on the TRACK questionnaire) and incremental costs per quality-adjusted life year (QALY) (based onEQ-5-DY).

### Pharmacotherapy

Use of asthma medication and antibiotics will be continually registered: drugs, dosage, and period of use. Parents will be asked to register this using the app “Qdot studies” developed by Maastricht University. This app is specifically designed to collect data for scientific studies through questionnaires. We will assess whether children with asthma get bronchodilators (for symptom relief) and maintenance use of ICS (in case of more severe symptoms and less controlled disease) according to (inter) national guidelines [29], and look for differences in proper treatment of asthma between intervention and control group.

### Exacerbation of wheezing

Parents will be asked to register all exacerbations of asthma-like symptoms. This is facilitated by the same mobile application as is used for registration of pharmacotherapy.

### Growth

Weight and height will be assessed in height standard deviation (SD) z-scores according to national growth data. Height velocity will be calculated.

### Asthma or transient wheeze diagnosis at 6 years

The final diagnosis of transient wheeze or asthma will be made by two paediatric pulmonologists after the clinical visit at the age of 6 years. These paediatric pulmonologists will be blinded for the probability diagnosis (if applicable) of the participants. The asthma diagnostic algorithm for children as published by the ERS taskforce in 2021 [38] will be used to establish this final diagnosis. A diagnosis of asthma is made in children with symptoms of asthma when at least two out of the following objective test results are abnormal: spirometry, BDR or FeNO. With respect to spirometry, an FEV_1_/FVC ≤ the lower limit of normal (LLN) or ≤ 80%, ór an FEV_1_ ≤ LLN or ≤ 80% of predicted should be considered abnormal. If this is the case, BDR testing will be performed and an increase in FEV_1_ of more than 12% and/or more than 200 mL following an inhalation of 400 µg salbutamol is considered as an abnormal test result. A FeNO value ≥ 25 ppb should be considered as an abnormal test result. In those patients in which spirometry is normal, but FeNO concentration is higher than 25 ppb, a direct bronchial challenge test using methacholine will be performed. A provocative concentration of methacholine of ≤ 8 ml/mL that results in a 20% drop in FEV_1_ should be considered as a positive test.

Statistical methods

General descriptive statistics will be applied to describe the baseline characteristics. Table 2 depicts the different study groups in which the main statistical analyses will be performed.

**Fig. 5:**
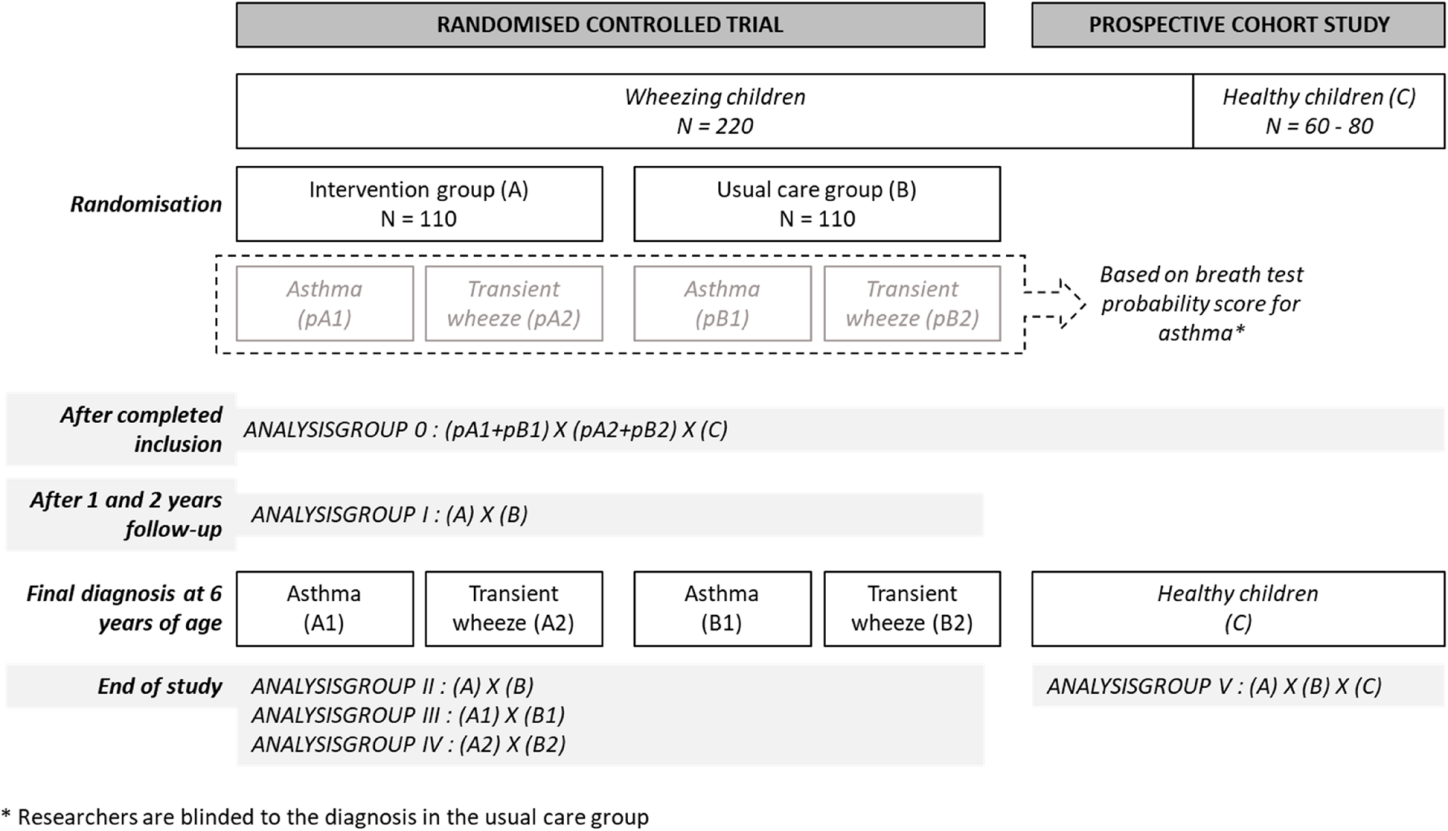
Corresponding analysis groups

**Table 2:** outcome parameters and corresponding outcome measures and methods of analysis (see Fig. 5 for corresponding analysis groups)

Outcome	Analysis group	Outcome measure	Methods of analysis
**Randomised Controlled Trial**			
*Primary*			
Difference in the percentage of well controlled asthma-like symptoms after 1-year follow-up	I	TRACK-score	Unpaired t-test / Mann Whitney-U test
*Secondary*			
Pharmacotherapy	I, II, III, IV	Cumulative ICS dosage (fluticasone equivalent)	Mann Whitney-U test
Growth retardation	I, II, III, IV	SD deviation from target height	Unpaired t-test
Amount of exacerbations	I, II, III, IV	Amount	Chi-square test
Amount of hospital admissions	I, II, III, IV	Amount	Chi-square test
Quality of life child	I, II, III, IV	EQ-5-DY by proxy	Unpaired t-test / Mann Whitney-U test
Quality of life caretaker	I, II, III, IV	EQ-5-DY	Unpaired t-test / Mann Whitney-U test
Lung function (tremoflo)	I, II, III, IV	R_5_, R_5-20_, X_5_, X_5-20_, AX, F_res_ (baseline and % change after salbutamol)	Unpaired t-test / Mann Whitney-U test
Lung function (spirometry)	II, III, IV	FEV_1,_ FVC, MEF50 (baseline and % of change after salbutamol)	Unpaired t-test / Mann Whitney-U test
Absence of school and work (parents)	I, II, III, IV	Number of days	Chi-square test
Healthcare resource use and –costs	I, II, III, IV		Unpaired t-test / Mann Whitney-U test
Costs outside healthcare	I, II, III, IV	Euro	Unpaired t-test / Mann Whitney-U test
Cost-effectiveness	I, II, III, IV	Societal cost per QALY and health care cost per additional child with control of asthma-like symptoms	Sensitivity and bootstrap analysis
**Prospective cohort study**			
*Primary*			
Identification of discriminative exhaled VOCs	V	n.a	n.a
Diagnostic value of the VOCs sensing techniques (GC-tof–MS, SIFT-MS) for a diagnosis of asthma or transient wheeze in preschool children*	V	Probability-score > 0.5 is defined as “asthma”	Sn, Sp, true positive and true negative rate
*Secondary*			
Identification of other potential discriminating biological parameters (such as allergic sensitisation, immunological markers, epigenetics, transcriptomics, microbiome) between asthma, transient wheeze, and healthy controls, and the subsequent identification of underlying disease pathways and relation to the discriminative VOCs in the exhaled breath	0, V	n.a	n.a

* based on algorithm

### Assessment of differences in asthma control between the intervention group and the usual care group in the percentage of well controlled asthma-like symptoms after 1- and 2-year follow-up.

The effect of the intervention on asthma control will be assessed by comparing the outcome measures between the intervention and the usual care group in the RCT. Asthma control will be scored using the validated TRACK questionnaire. Differences of the continuous outcome measure between the intervention group and the usual care group will be tested for significance with the unpaired t-test for normally distributed parameters and the Mann Whitney-U test in case of a not-normal distribution.

### Assessment of improvement in health gain and costs of care with the application of the breath test in wheezing preschool children

The effect of the intervention will be assessed by comparing the outcome measures between the intervention and the usual care group in the RCT. Dichotomous parameters will be tested with the chi-square test. Continuous variables will be tested for significance with the unpaired t-test for normally distributed parameters and the Mann Whitney-U test in case of a not-normal distribution.

Total treatment costs will be calculated by multiplying resource use with the costs per unit. Resource use (visits to the general practitioner or specialist, emergency visits, hospital admission, lung function tests and other diagnostic procedures, the breath test, (over-the-counter) medication, and lost work days by parents due to sickness of the child) will be obtained from a specially designed questionnaire with a recall period of three months. The parents will fill out this questionnaire at baseline and at 3 months interval during the follow-up. Sources for valuation of the costs will be cost-prices of the Dutch manual for costing and cost-prices from the Dutch pharmacotherapeutic compass [45, 47–49] (reference data 2022). If necessary, local hospital cost-prices will be used, which are largely based on integral cost-prices from the Dutch hospitals [50]. Absence of work reported by the parents will be calculated by using the friction cost method, which is recommended by the Dutch manual for costing [47, 48].

The cost-effectiveness analysis from the healthcare perspective will be based on symptom control according to the TRACK questionnaire at 1 year follow-up (primary outcome measure). The cost-effectiveness analysis from the societal perspective will be based on the EQ-5D-Y. The EQ-5D-Y will be completed at baseline and at 3 months intervals during the follow-up, and will be filled out by one of the parents.

A cost-effectiveness analysis will be performed from a societal and healthcare perspective with a time horizon of 2 years. Incremental cost-effectiveness ratios will be calculated as societal cost per QALY (societal perspective) and health care cost per additional child with control of asthma-like symptoms (healthcare perspective). Standard sensitivity- and bootstrap analysis will be performed to address uncertainty regarding costs and cost-effectiveness outcomes. Cost-effectiveness acceptability curves will be constructed reflecting the probability that the diagnostic breath test is cost-effective for a range of threshold values. Costs and effects after one year will be discounted at 4.0% and 1.5% respectively, according to the Dutch guidelines for health economic evaluation [45, 48].

All outcome parameters and corresponding outcome measures and methods of analysis are listed in Table 2. The large number of patients (*n* = 220) allows better representation of specific subgroups of specific age, background (primary or secondary/tertiary care), genetic predisposition, and geographic area. All these parameters will be measured and included in the multivariable analysis in order to assess possible influences on outcome. Intention-to-treat analyses will be applied. We will execute two subgroup analyses: one for age and one for level of care (first-line versus secondary/tertiary care). Two-sided *p*-values < 0.05 (with correction for multiple testing) will be regarded as statistical significant.

### Assessment accuracy of the VOC sensing techniques (GC-tof–MS, SIFT-MS)

The primary outcome of the prospective cohort study is assessed in two ways, namely by comparing the VOCs data of the two VOCs-sensing techniques (GC-*tof*–MS and SIFT-MS) at inclusion of all participating children (both wheezing preschool children and healthy controls) to the final diagnosis at six years of age, and by comparing the VOCs-data of the SIFT-MS at the baseline visit to the results of the gold standard (GC-*tof*–MS) at the baseline visit. The sensitivity, specificity, positive predictive value and negative predictive value for a diagnosis of asthma of the two different VOCs sensing techniques (GC-*tof*–MS and SIFT-MS) will be determined with corresponding 95% confidence intervals. Statistical analysis of the volatilome will be performed by current published standards on data analysis for VOCs analysis in breath [51]. The extensive VOCs data derived from the mass spectrometry analyses will be implemented in an algorithm by using mathematical models for analysis of sensor signals (for instance neuronal networks, random forest, support vector machine, or principle component analysis) as described previously [37].

### Assessment of pathogenic pathways in the early development of asthma

The secondary outcomes of the prospective cohort study will be assessed by an integrative omics approach. The high dimensional multi-omics data require advanced statistical analyses. We will use machine learning and multi-variate statistical approaches (such as elastic net and weighted gene co-expression network analysis) that have been proven successful in analysing complex, multilevel datasets. Also, mechanistic models that provide a detailed understanding of biological networks will be used. Such models (e.g. Recon2) provide a comprehensive ‘reconstruction’ of the human biology and can be used to infer causality by integrating several layers of information (e.g. gene expression, metabolomics, and microbiomics) [52, 53].

### Data monitoring and management

Despite the fact that this study will be conducted in a paediatric population, the implementation of a Data Management Committee is not indicated. This decision was mainly based on the fact that the intervention of the study (probability diagnosis based on the breath test) provides caregivers and treating physicians with disease-specific treatment recommendations, but does not obligate them to adhere to a specific treatment protocol.

The ADEM2 database is developed by the Clinical Trial Centre Maastricht (CTCM) in collaboration with MEMIC (centre for data and information management at the Faculty of Health, Medicine and Life Sciences of Maastricht University and MUMC +). CTCM is one of the leading Academic Research Organizations (ARO’s) in the Netherlands, and provides services to facilitate research, including the set-up of databases that meet the highest quality standards and newest guidelines. The data are collected by using the CASTOR application, an electronic tool that is adapted to the ADEM2 requirements. Data are collected by using the CTCM coding, which can easily be supplemented with an International coding (for example SNOMED). Metadata is included in the application of the electronic Case Report File (CRF) (CASTOR). This includes, for example, the units that are used, but also the coding of the variables. Data is securely stored for 15 years at CTCM. CASTOR provides export to various data formats, including SPSS, SAS, XML, CSV and Excel. By using CASTOR, the data collected in ADEM2 are according to the FAIR (Findable, Accessible, Interoperable and Reusable) criteria. By using clear coding with metadata and having the possibility to export the data to different formats, the data are interchangeable and reusable.

### Auditing

Independent review of core trial processes and documents will be executed through periodic, scheduled, on-site, monitoring visits. Processes such as participant enrolment, consent, eligibility, allocation to study groups, adherence to trial interventions, policies to protect participants, and completeness and accuracy of data collection will be reviewed during these visits. Audits will be conducted at all measurement sites that participate in this multi-centre trial.

### Ethics

NL64912.068.18 (11^th^ of April 2019). The study will be conducted according to the principles of the Declaration of Helsinki (October 2013) and in accordance with the Dutch Medical Research Involving Human Subjects Act (WMO). Ethical approval is obtained from the Dutch National Medical Ethical Committee (CCMO). In case of substantive protocol amendments, they will be reviewed by the Dutch National Medical Ethical Committee.

Both wheezing children and healthy children will be invited through an invitation letter combined with the Subject Information (see section on recruitment for more details). Parents are encouraged to contact the study team in case of any questions. We will ask the parents, if they decide to participate in the study to fill in the informed consent form (by both parents) and send it back to us.

This study is registered by the Netherlands Trial Register (NTR) (www.trialregister.nl, registration number NL7336).

### Confidentiality and access to data

Data will get a code and will be handled confidentially in accordance with the General Data Protection Regulation (GDPR). The code is based on an unique participant number, the disease status (healthy versus wheezing participants), and the centre and region where the participant comes from. Our secured, web-based study management system “Ldot” will be used to link the data to the subject in case it is necessary to trace data to an individual subject. The key to the code will be safeguarded by the investigator (in accordance with www.fmwv.nl). The research nurse, the principal investigator, the investigator, the IGJ (‘inspectie gezondheidszorg en jeugd’) and the monitor will have access to the data.

### Public disclosure and publication policy

We will comply with the ‘CCMO statement publication policy’. Positive as well as negative findings will be published. After completion, the study results will be made known to the CCMO and the public.

### Biological specimens

All biological specimens (faeces, blood, nasopharyngeal swabs, buccal swabs for DNA extraction, nasal swabs) will be coded and stored in the BioBank Maastricht UMC + and the UMCG for 10 years. These specimens will be used for the current trial and may be used for future research questions or analyses of new biomarkers. The data of the BioBank (such as the project number, respondent number, numbers, amount of samples available, and information about the quality) will be stored in a fully automatic web-based BioBank Information System (BIS).

## Discussion

In this study protocol we described the assessment of the three main objectives of the ADEM2 study: with respect to the first objective, a multicentre RCT will be performed to assess the potential gain in health and reduction of health care related costs by means of a proper early diagnosis through the breath test in wheezing preschool children. Parallel to the RCT, a longitudinal observational cohort study will be executed to unravel early and important pathogenic mechanisms of asthma and transient wheeze (second objective), and to assess the diagnostic potential of alternative VOCs sensing techniques besides GC-tof–MS as well as other multi-omics measurements (third objective).

### Relevance and societal and clinical impact

The potential societal and clinical impact of the diagnostic tool for the children and the relevance of the project is substantial. By means of the breath test, it will become possible to deliver high qualitative care to the large group of vulnerable children with asthma-like symptoms, which will be more effective, safe, early and on time, and customised based on the individual results of the children. That will be a great step forward. The development of the current non-invasive breath test (GC-*tof*–MS) into a smart, feasible, relatively cheap, and also non-invasive device will be a solution for a large clinical problem in a substantial group of young children.

### Methodological issues

We decided to choose a RCT design for the following reasons: 1) to assess the full potential of the breath test in health gain and costs of care, a comparative study design is needed; 2) the breath test is not standard care yet; 3) in the usual care group, it is not unethical to provide the test result in a later phase, eventually all children/parents will benefit from the breath test result; 4) a safety rule will be applied in the usual care group so that parents/treating doctors can get the result of the breath test in an urgent situation (e.g. severe exacerbations, hospital admissions).

At first, we did not expect any problems with the feasibility of patient recruitment based on the high prevalence of children with asthma-like symptoms and our experiences during the first ADEM study. However, the outbreak of the SARS-CoV-2 pandemic in 2020 significantly affected the recruitment of participants. In the beginning of the pandemic, we were not allowed to perform research anymore by the Board of Directors of our hospitals. In a later phase, less preschool children with wheezing symptoms presented themselves at the outpatient clinic, emergency departments, and primary care facilities than before the crisis, which probably was the consequence of preventive measures taken (e.g. the lock-downs and temporary closure of schools and day-care facilities). As most of our patients were selected during and after the pandemic, this influenced intervention and control group equally.

We do not expect problems with drop-outs during the study because the breath test and the questionnaires are non-invasive, and the parents, children and treating doctors are highly motivated. Moreover, the ADEM study had a comparable design and burden with only 2% loss to follow-up [22]. We will limit the expectations of the parents and health-care workers and emphasize that the breath test has not a 100% reliability. We will keep the contrast between intervention and control group as large as possible by continuous training and instruction of treating doctors and centres. One potential pitfall of the RCT may be a small contrast between intervention and usual care group. Doctors are not used to get a reliable diagnosis of asthma in pre-schoolers and may not act appropriately on the result of the breath test. Moreover, we cannot exclude that at least some children with transient wheeze may benefit from treatment with ICS, which may diminish the contrast between intervention and control group as well.

### Feasibility of implementation

The current breath test is based on GC-*tof–*MS, the gold standard for breath analysis. However, the complex analytical method GC-*tof*–MS is time-consuming, expensive, and requires a lot of expertise. As a consequence, the implementation of this breath test in daily clinical practice will be challenging. Therefore, in the ADEM2 project, we aim to further develop the breath test into a small, reliable, and fast, POC breath test. This one-stop breath analyser must fulfil the quality requirements such as: easy to perform for children, parents and laboratory workers, high feasibility and very good reliability, and fast results within hours to days.

It is our goal to implement a breath test at all levels of care: from primary care (general practitioner offices) to regional hospitals (with general paediatric care) and academic hospitals (with paediatric pulmonologists). The breath test will certainly help to overcome the problem of the absence of a diagnostic test in the large group of children with asthma-like symptoms.

We expect that the feasibility of the implementation of results is high because patients and health care professionals recognise the clinical problem, ask for a diagnostic test, and are involved in this proposal. The topic has been selected by the Lung Foundation Netherlands as one of the important research themes.

### Health gain and cost saving

The health gain will directly arise as a consequence of a higher proportion of children with asthma control. Furthermore, a considerable annual cost-saving might occur because of reduced referrals and hospital visits after a diagnosis of transient wheeze is established. Not included in the calculations are positive quality of life (QoL) effects due to reductions in side-effects of asthma medication in children with transient wheeze, and broader QoL effects due to better asthma control and less exacerbations, which will impact quality-adjusted-life-years but is hard to estimate. Therefore, both the overall health effect and cost savings are expected to be larger than calculated above. As a consequence, the cost-efficiency of health care to this large group of children will substantially improve. Moreover, by means of the breath test, the cost-effectiveness of pharmacotherapy in this group of children will increase. The breath test will result in concrete and significant improvements for daily clinical care: by using the test, a substantial gain in health outcome parameters as well as in costs of care will occur.

### Insights in pathogenic mechanisms in the early development of asthma

With our multi-omics approach we expect to unravel important pathogenic pathways in the early development of asthma and transient wheeze. We will apply genomics, transcriptomics of blood and nasal epithelium, microbiomics, epigenetics, and metabolomics to establish an integral pathogenic mechanisms for the early development of asthma.

In the ADEM-study we found an interaction between bacterial colonisation of the upper airways, genetic variants in the *TLR*s and *CD14* genes, and the development of asthma at age 6 years [23]. In the same cohort (and replicated in an independent birth cohort) a negative association of the CG/GG-genotype of rs528557 in the *ADAM33* gene with childhood asthma was found, confirming that genetic variation in the *ADAM33* gene may be implicated in the progression of wheeze into childhood asthma [24]. In an integrative genomic approach, data suggested that *ICAM-1* was likely to be involved in the development of childhood asthma [25].

Since the ADEM-study, multi-omics techniques extended and improved substantially, which increase the possibility to identify basic pathogenic mechanisms in ADEM2. Once fundamental pathways have been revealed, potential new therapies can be developed and tested, which hopefully can prevent the early development of asthma in wheezing preschool children.

## Conclusion

In summary, the ADEM2 project covers 3 main areas. First, a multicentre RCT will be performed to assess the hypothesised gain in health and reduction of health care related costs by means of a proper early diagnosis using the breath test in wheezing preschool children. Second, the longitudinal observational cohort study is set up to unravel early and important pathogenic mechanisms of asthma and transient wheeze. And third, this project facilitates the development and assessment of the diagnostic potential of alternative VOCs sensing techniques besides GC-tof–MS as well as other multi-omics measurements.

## Data Availability

Not applicable.

## Abbreviations

API: Asthma Predictive Index
ATS: American Thoracic Society
AUC: Area under the ROC Curve
BDR: Bronchodilator responsiveness
CPAP: Continuous Positive Airway Pressure
CRF: Case Report File
ERS: European Respiratory Society
FACS: Fluorescence-activated single cell sorting
FeNO: Fractional exhaled Nitric Oxide
FEV1: Forced expiratory volume in one second
FVC: Forced vital capacity
GC-MS: Gas chromatography mass spectrometry
GC-tof-MS: Gas chromatography–time-of-flight–mass spectrometry
GDPR: General Data Protection Regulation
HRQoL: Health-related quality of life
ICS: Inhaled corticosteroids
IgE: Immunoglobulin E
m/z: Mass-to-charge ratio
MEF50: Maximal expiratory flow at 50% FVC
MUMC: Maastricht University Medical Center
PBMC: Peripheral blood mononuclear cell
POC: Point-of-care
QALY: Quality-adjusted life year
QoL: Quality of Life
RCT: Randomised controlled trial
RUMC: Radboud University Medical Center
SD: Standard Deviation
SIFT-MS: Selected ion flow tube mass spectrometry
SOP: Standard Operating Procedure
TLR: Toll-like receptor
UMCG: University Medical Center Groningen
UTM: Universal Transport Medium
VAS: Visual analogue scale
VOC: Volatile Organic Compound
